# Structural changes of hip osteoarthritis using magnetic resonance imaging

**DOI:** 10.1186/s13075-014-0466-4

**Published:** 2014-10-11

**Authors:** Andrew J Teichtahl, Yuanyuan Wang, Sam Smith, Anita E Wluka, Graham G Giles, Kim L Bennell, Richard O’Sullivan, Flavia M Cicuttini

**Affiliations:** Department of Epidemiology and Preventive Medicine, School of Public Health and Preventive Medicine, Monash University, Alfred Hospital, Melbourne, VIC 3004 Australia; Baker IDI Heart and Diabetes Institute, Commercial Road, Melbourne, VIC 3004 Australia; Centre for Epidemiology and Biostatistics, Melbourne School of Population and Global Health, The University of Melbourne, Carlton, VIC 3053 Australia; Cancer Epidemiology Centre, Cancer Council Victoria, Melbourne, VIC 3004 Australia; Centre for Health, Exercise and Sports Medicine (CHESM), Department of Physiotherapy, The University of Melbourne, Victoria, Australia; MRI Department, Healthcare Imaging Services, Epworth Hospital, Richmond, VIC 3121 Australia; Department of Medicine, Central Clinical School, Monash University, Melbourne, VIC 3004 Australia

## Abstract

**Introduction:**

Few data are available concerning structural changes at the hip observed by magnetic resonance imaging (MRI) in people with or without hip osteoarthritis (OA). The aim of this study was to compare cartilage volume and the presence of cartilage defects and bone marrow lesions (BMLs) in participants with and without diagnosed hip OA.

**Methods:**

Femoral head cartilage volume was measured by MRI for 141 community-based persons with no diagnosed hip OA, and 19 with diagnosed hip OA. Cartilage defects and BMLs were regionally scored at the femoral head and acetabulum.

**Results:**

Compared with those without diagnosed hip OA, people with diagnosed hip OA had less femoral head cartilage volume (1763 mm^3^ versus 3343 mm^3^; p <0.001) and more prevalent cartilage defects and BMLs (all p ≤0.05) at all sites other than the central inferomedial region of the femoral head. In those with no diagnosed hip OA, cartilage defects in the anterior and central superolateral region of the femoral head were associated with reduced femoral head cartilage volume (all p ≤0.02). Central superolateral BMLs at all sites were associated with reduced femoral head cartilage volume (all p ≤0.003), with a similar trend occurring when BMLs were located in the anterior region of the hip (all p ≤0.08).

**Conclusions:**

Compared with community-based adults with no diagnosed hip OA, people with diagnosed hip OA have less femoral head cartilage volume and a higher prevalence of cartilage defects and BMLs. For people with no diagnosed hip OA, femoral head cartilage volume was reduced where cartilage defects and/or BMLs were present in the anterior and central superolateral regions of the hip joint. Cartilage defects and BMLs present in the anterior and central superolateral regions may represent early structural damage in the pathogenesis of hip OA.

## Introduction

Osteoarthritis (OA) of the knee and hip are common clinical problems and major causes of pain, disability and cost to the community through joint replacement surgery. Through non-invasive assessment of the knee using magnetic resonance imaging (MRI), a large body of research has examined knee structures from health through to disease [1]. Such data have contributed to better understanding early pathological changes in the knee joint, and how these relate to symptoms and progression of knee OA. Structural abnormalities such as bone marrow lesions (BMLs) and focal cartilage defects have been shown to predate cartilage volume loss and subsequent radiographic joint disease, as well as joint replacement in knee OA [2–6]. Such advances have resulted in novel, albeit experimental, preventive and therapeutic approaches to knee OA, whereby modification of these lesions can be assessed as an outcome for early disease progression [7–9]. Despite the significant burden of hip OA, few studies have examined the structural changes at the hip in OA, and how these differ from those without diagnosed disease. This is primarily because it has been challenging to develop MRI protocols that adequately visualise the deeply located and complex structure of the hip joint in three-dimensional analyses.

There is clear epidemiological evidence that knee and hip OA result from different pathogenic processes. For example, whereas obesity and the metabolic syndrome are major risk factors for the structural changes in knee OA, neither obesity nor the metabolic syndrome has been shown to be a consistent or strong risk factor for hip OA [10,11]. Therefore, the causes and correlates of structural changes in knee OA cannot simply be extrapolated to the structural changes in hip OA. Most studies examining structural changes in hip OA have focussed on radiographic assessment but, as at the knee, a major limitation is that radiographs do not capture the early structural changes that may occur before joint symptoms are recognised. By the time early radiographic joint space narrowing of the hip is detected, 13% of femoral head cartilage volume has already been lost [12]. Sparse data are available examining structural changes in the hip joint in individuals without diagnosed hip OA, and whether these changes may be associated with hip OA. Moreover, no data are available on the location specific significance of structural abnormalities at the hip. At the knee, a disproportionate amount of damage is located in the medial, rather than the lateral compartment. Attempts have been made to characterise regional structural damage at the hip, but these have been limited by small sample sizes and have not provided any regional prevalence data [13,14].

As MRI has enabled a better understanding of the impact of knee OA from early through to established joint disease, we performed a cross-sectional MRI study to examine the prevalence and interrelationships of MRI-determined structural abnormalities in people with and with no diagnosed hip OA in a region-specific manner.

## Methods

### Participants

#### Participants with no diagnosed hip OA

A total of 141 community-based adults with no diagnosed hip OA were recruited between 2009 and 2010 from the Melbourne Collaborative Cohort Study (MCCS), a prospective cohort study of 41,514 residents of Melbourne, Australia, aged 40 to 69 years at MCCS inception (1990 to 1994), examining healthy ageing [15]. Participants were eligible for the current study if they were aged 50 to 85 years without any of the following exclusion criteria: diagnosis of hip OA made by a medical or allied health professional; significant hip pain lasting for >24 hours in the last 5 years; a previous hip injury requiring non-weight bearing treatment for >24 hours or surgery (including arthroscopy); a malignancy; a history of any form of arthritis diagnosed by a medical practitioner; or a contraindication to MRI including pacemaker, metal sutures, presence of shrapnel or iron filings in the eye, or claustrophobia.

#### Participants with diagnosed hip OA

For comparative purposes, 19 people aged 50 to 79 years who fulfilled the American College of Rheumatology (ACR) classification for a diagnosis of clinical hip OA [16] were recruited between 2009 and 2010 using a convenience sample by advertising in the local community. Participants with a diagnosis of hip OA had a hip radiograph performed on their symptomatic joint to confirm the presence of radiographic hip OA (Kellgren-Lawrence grade ≥2) prior to MRI. Any person was excluded if they had any malignancy or contraindication to MRI. The study was approved by Human Research Ethics Committees of The Cancer Council Victoria, Monash University, and University of Melbourne. All participants gave written informed consent.

### Anthropometric data

Anthropometric data were collected at the time of MRI (2009 to 2010). Height was measured using a stadiometer and weight using electronic scales. Body mass index (BMI) (weight/height^2^, kg/m^2^) was calculated.

### MRI measurements

Between 2009 and 2010, participants with no diagnosis of hip OA had an MRI performed on their dominant hip, defined by the leg used to kick a ball (89% right-sided), and those with a diagnosis of hip OA had an MRI of their affected hip. Hips were imaged on a 3.0-T whole body magnetic resonance unit (Siemens, Verio, Siemens Medical, Germany) using a phased array flex coil. Sagittal images were obtained using a T_2_-weighted fat-suppressed 3-dimensional gradient-recalled acquisition sequence in the steady state (repetition time 14.45 msec, echo time 5.17 msec; flip angle 25°, slice thickness 1.5 mm, field of view 16 cm, pixel matrix 320 × 320, acquisition time 7 minutes 47 sec, and 1 acquisition). Coronal images were obtained using a fat saturation, proton density, spin echo acquisition sequence (repetition time 3,400 msec, echo time 64 msec, flip angle 90°, slice thickness 3 mm, field of view 16 cm, pixel matrix 256 × 256, acquisition time 5 minutes 26 sec, and 1 acquisition). The same protocol at the same location, using the same MRI scanner occurred regardless of whether a participant had diagnosed hip OA or not. A musculoskeletal radiologist with over 15 years experience using structural outcomes determined by MRI in epidemiological studies supervised and independently monitored measurements. One observer, trained by the radiologist was responsible for measuring one structural outcome (for example, cartilage volume, cartilage defects or BMLs). Each observer was also required to assess their designated structural measure in duplicate, at least one week apart and blinded to their previous assessment and characteristics of the participants.

Femoral head cartilage volume was measured from T_2_-weighted sagittal images using the software Osiris (version 4.19; Geneva University Hospital, Geneva, Switzerland) as previously described [12]. The volume of the femoral head cartilage was isolated from the total volume by manually drawing disarticulation contours around the cartilage boundaries on each image section. These data were then resampled by bilinear and cubic interpolation for the final 3-dimensional rendering. The volume of the femoral head cartilage was determined by summing all the pertinent voxels within the resultant binary volume. Femoral head cartilage volume was measured in duplicate with at least a 1-week interval by one trained observer. The coefficient of variation (CV) was 2.5% [12]. The intra-observer reproducibility assessed by intra-class correlation coefficient (ICC) was 0.99.

**Figure 1:**
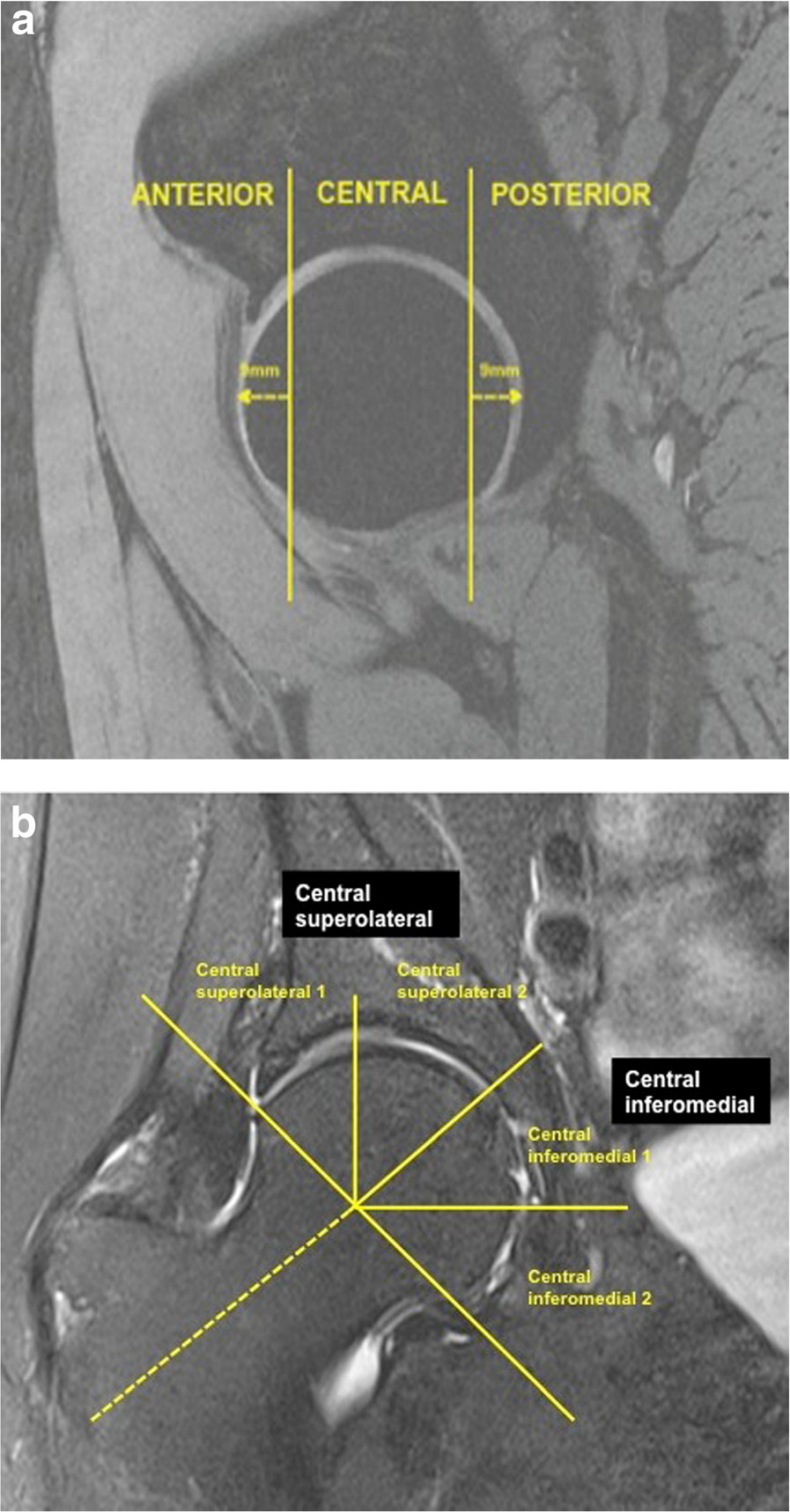
**Regional zones of the hip joint. (a)** Sagittal image depicting the anterior, central and posterior regions; **(b)** coronal image depicting the central superolateral and inferomedial regions.

The hip joint was divided into three regions: central, anterior and femoral. The anterior and posterior regions were assessed in the sagittal plane and corresponded to the first and last three coronal slices (9 mm) (Figure 1a). The area in between the anterior and posterior region was termed the central region. The central region was further subdivided in the coronal plane (Figure 1b). The intersection of the axis of the femoral head and neck was considered to be the midpoint of the region, with the axis of the femoral neck used to demarcate the central superolateral from the central inferomedial region. The central superolateral and inferomedial regions were then further subdivided for exploratory analyses. The division of anterior, central and posterior regions was adapted from methods used in previously published works [13,14].

Femoral head and acetabular cartilage defects were assessed from proton density coronal images and confirmed on sagittal imaging for the central region, and from the sagittal imaging only for the anterior and posterior regions. The presence of cartilage defects was defined as loss of cartilage thickness of more than 50% which was shown on at least two consecutive slices. One trained observer, who was blinded to participant’s characteristics, assessed the presence of cartilage defects for each participant in duplicate, at least one week apart. The ICC for intra-observer reproducibility was 0.72. Cartilage defects were only assessed at the central superolateral region of the acetabulum because there was no cartilage at the inferomedial region of the acetabulum, an observation corroborated both by a previous MRI study [13] and at arthroscopy [17].

Femoral head and acetabular BMLs were assessed on fat-suppressed proton density coronal images in the regions as described for cartilage defects. BMLs were defined as areas of increased signal intensity in subchondral regions in the femoral head and acetabulum. The presence of a BML was defined if it appeared on two or more adjacent slices and was visible both in the sagittal and coronal planes. One trained observer, who was blinded to participants' characteristics, assessed the presence of BMLs for each participant in duplicate, at least one week apart. The ICC for intra-observer reproducibility was 0.94.

The sagittal image closest to the centre of the femoral head was used to measure the femoral head bone area. It was measured by drawing contours around the femoral head bone, and area calculated automatically by the Osiris program as an indicator of bone size. Femoral head bone area was measured by one trained observer with random crosschecks performed by a second observer. The CV was 1.1% [12]. The ICC for inter-observer reproducibility was 0.99.

### Statistical analyses

Between-group differences for people with and without diagnosed hip OA were assessed using either chi-square analysis for dichotomous variables or the independent samples *t*-test when the variable was continuous. Multiple linear regression analysis was used to determine the relationship between femoral head cartilage volume and other hip structural abnormalities in participants without clinical hip OA. A *P*-value <0.05 (two-tailed) was regarded as statistically significant. All analyses were performed using the SPSS statistical package (standard version 20.0 SPSS, Chicago, IL, USA).

## Results

Participant characteristics are shown in Table 1. People with hip OA were younger than the non-OA cohort (59.2 years versus 66.8 years; *P* <0.001) and had smaller femoral head cartilage volume (1,763 mm^3^ versus 3,343 mm^3^; *P* <0.001) (Table 1).

**Table 1:** **Participant characteristics**

	**Diagnosis of osteoarthritis**	**No diagnosis of osteoarthritis**	***P*** **-value** ^**1**^
	**(n = 19)**	**(n = 141)**	
Age, years	59.2 (7.6)	66.8 (7.3)	<0.001^2^
Gender, % female	57.9	55.6	0.85
Body mass index, kg/m^−2^	27.2 (4.8)	27.6 (4.8)	0.74^2^
Kellgren-Lawrence grade			
2	12 (63)		
3	6 (32)		
4	1 (5)		
**Hip structures on magnetic resonance imaging**			
Femoral head cartilage volume, mm^3^	1763 (321)	3343 (808)	<0.001^2^
Femoral head bone area, mm^2^	1651 (305)	1610 (255)	0.58^2^
**Cartilage defects, number (%)**			
**Femoral head**			
Central superolateral	17 (89.5)	45 (31.9)	<0.001
Central inferomedial	10 (52.6)	67 (47.5)	0.68
Anterior	12 (63.2)	5 (3.5)	<0.001
Posterior	14 (73.7)	25 (17.6)	<0.001
**Acetabular**			
Central superolateral	17 (89.5)	37 (26.2)	<0.001
Anterior	18 (94.7)	26 (18.3)	<0.001
Posterior	15 (78.9)	50 (35.2)	<0.001
**Femoroacetabular**			
Central superolateral	18 (94.7)	64 (45.1)	<0.001
Anterior	18 (94.7)	27 (19)	<0.001
Posterior	16 (84.2)	57 (40.1)	<0.001
**Bone marrow lesions, number (%)**			
**Femoral head**			
Central superolateral	10 (52.6)	7 (5.0)	<0.001
Central inferomedial	6 (31.6)	6 (4.3)	<0.01
Anterior	7 (36.8)	2 (2.1)	<0.001
Posterior	5 (26.3)	4 (2.8)	<0.001
**Acetabular**			
Central superolateral	13 (68.4)	22 (15.6)	<0.001
Central inferomedial	2 (10.5)	3 (2.1)	0.05
Anterior	13 (68.4)	28 (19.7)	<0.001
Posterior	11 (57.9)	18 (12.7)	<0.001
**Femoroacetabular**			
Central superolateral	14 (73.7)	27 (19.0)	<0.001
Central inferomedial	6 (31.6)	8 (5.6)	<0.001
Anterior	14 (73.7)	29 (20.4)	<0.001
Posterior	13 (68.4)	20 (14.1)	<0.001

Results displayed as mean (±SD) unless otherwise stated. All central regions results are the combined prevalence of regions 1 and 2. ^1^ *P*-value for differences between groups (chi-square test unless otherwise stated). ^2^Independent samples *t*-test.

**Figure 2:**
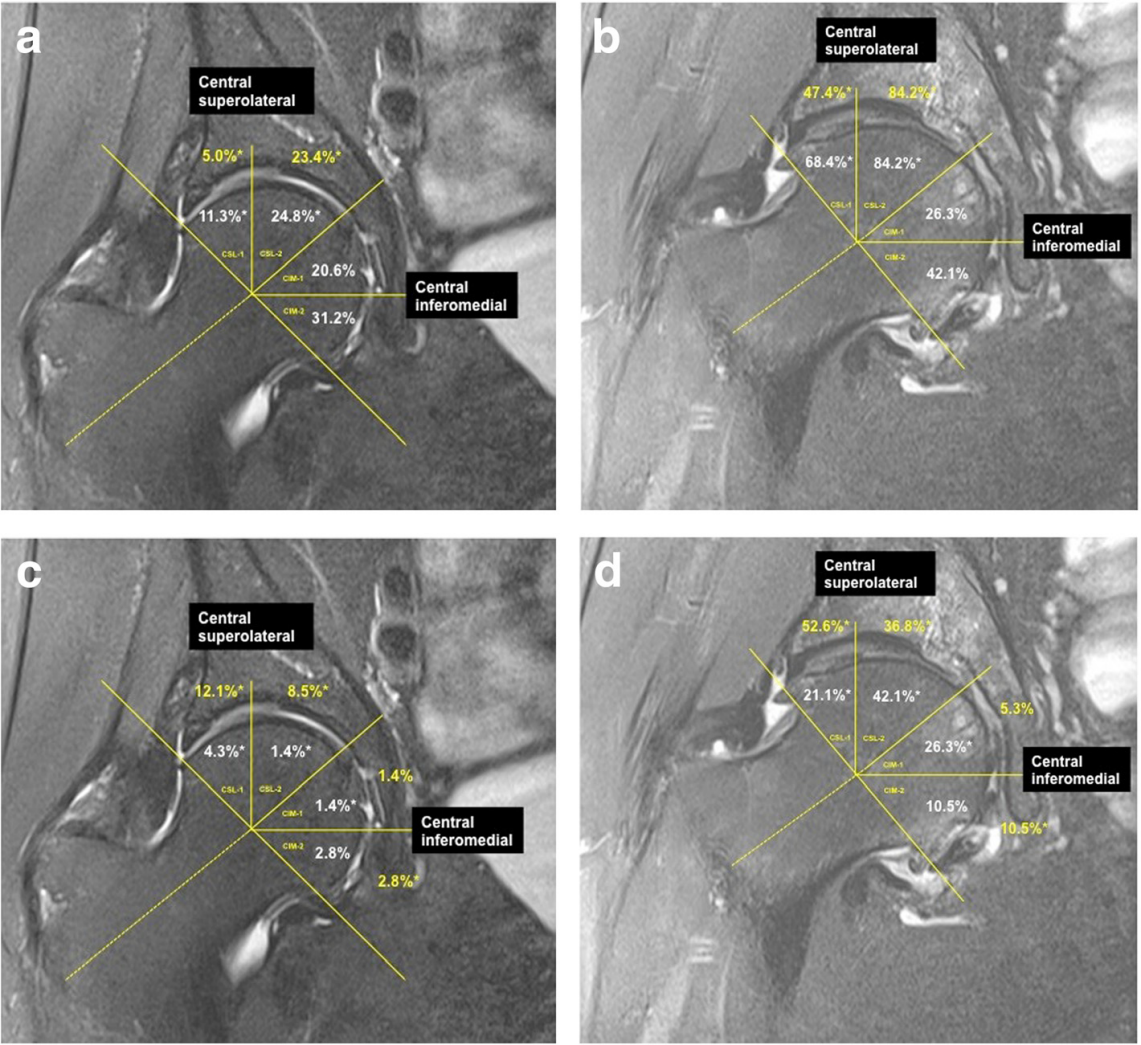
**Prevalence of cartilage defects (a and b) and bone marrow lesions (c and d) in people with and without diagnosed hip osteoarthritis (OA).** *P <0.05. Note: without OA should line up with columns **a** and **c**, while with OA lines up with **b** and **d** in a 2 × 2 set-up.

Figure 2 depicts the prevalence of central cartilage defects and BMLs for people both with and without diagnosed hip OA. Cartilage defects and BMLs were more prevalent in people with, compared to those without diagnosed hip OA in all regions (all *P* ≤0.05), excluding cartilage defects in central inferomedial regions 1 and 2 of the femoral head. When these results were adjusted for age, gender and BMI, results were unchanged (data not shown).

As shown in Table 1, the prevalence of combined central superolateral and inferomedial cartilage defects and BMLs was significantly greater in people with hip OA (all *P* <0.001), excluding the combined central inferomedial region of the femoral head (*P* = 0.68). The prevalence of anterior and posterior cartilage defects and BMLs were significantly greater in people with, than those without diagnosed hip OA (all *P* <0.001) (Table 1). When these results were adjusted for age, gender and BMI, results were unchanged (data not shown). Moreover, we also examined whether the difference in femoral head cartilage volume between groups was attributable to variation in the baseline variables for age, gender, BMI and femoral head bone area. After adjusting for these factors, femoral head cartilage volume remained significantly lower in people with diagnosed hip OA (estimated marginal means: 1656 mm^3^ versus 3357 mm^3^, *P* <0.001).

As cartilage volume was significantly reduced in participants with diagnosed hip OA, the associations between femoral head cartilage volume and the presence of cartilage defects and BMLs were examined in people without diagnosed hip OA (Tables 2 and 3). The small sample size precluded such analyses in that subgroup of people with diagnosed hip OA (n = 19).

**Table 2:** **The associations between hip cartilage defects and femoral head cartilage volume in people with no diagnosed hip osteoarthritis (n = 141)**

	**Univariate**	***P*** **-value**	Multivariate*	***P*** **-value**
	**β (95% CI)**		**β (95% CI)**	
**Femoral head**				
Central superolateral	−184 (−471, 104)	0.21	−283 (−460, −106)	0.002
Central inferomedial	−35 (−305, 234)	0.80	−11 (−180, 158)	0.90
Anterior	−357 (−1083, 371)	0.33	−800 (−1246, −354)	0.001
Posterior	−80 (−432, 273)	0.66	−198 (−421, 25)	0.08
**Acetabular**				
Central superolateral	−117 (−423, 188)	0.45	−35 (−228, 158)	0.72
Anterior	−320 (−664, 23)	0.08	−259 (−478, −41)	0.02
Posterior	251 (−27, 530)	0.08	−151 (−336, 33)	0.11
**Femoroacetabular**				
Central superolateral	−201 (−469, 67)	0.14	−153 (−324, 17)	0.08
Anterior	−332 (−671, 6)	0.05	−277 (−491, −63)	0.01
Posterior	138 (−135, 411)	0.32	−144 (−321, 32)	0.11

*Adjusted for age, gender, body mass index and femoral head bone area. All central results are combined prevalence of regions 1 and 2.

**Table 3:** **The associations between hip bone marrow lesions and femoral head cartilage volume in people with no diagnosed hip osteoarthritis (n = 141)**

	**Univariate**	***P*** **-value**	Multivariate*	***P*** **-value**
	**β (95% CI)**		**β (95% CI)**	
**Femoral head**				
Central superolateral	−106 (−727, 515)	0.74	−610 (−990, −229)	0.002
Central inferomedial	−406 (−1071, 259)	0.23	32 (−405, 469)	0.89
Anterior	−247 (−1182, 687)	0.60	−684 (−1260, −108)	0.02
Posterior	−553 (−1360, 255)	0.18	−380 (−891, 131)	0.14
**Acetabular**				
Central superolateral	−125 (−496, 246)	0.51	−250 (−577. -123)	0.003
Central inferomedial	−646 (−1575, 283)	0.65	−230 (−821, 360)	0.44
Anterior	−241 (−578, 95)	0.16	−189 (−400, 21)	0.08
Posterior	110 (−294, 514)	0.59	−16 (−274, 242)	0.90
**Femoroacetabular**				
Central superolateral	−59 (−401, 284)	0.74	−373 (−583, −164)	0.001
Central inferomedial	−468 (−1046, 110)	0.11	−80 (−452, 292)	0.67
Anterior	−266 (−597, 64)	0.11	−207 (−415, 1)	0.05
Posterior	77 (−310, 463)	0.70	−50 (−296, 196)	0.69

*Adjusted for age, gender, body mass index and femoral head bone area. All central results are combined prevalence of regions 1 and 2.

The associations between hip cartilage defects and femoral head cartilage volume in people with no diagnosed hip OA are shown in Table 2. After adjusting for age, gender, BMI and femoral head bone area, reduced femoral head cartilage volume was associated with the presence of cartilage defects at the anterior femoral head (β −800 mm^3^, 95% CI −1246 to −354 mm^3^, *P* = 0.001), anterior acetabulum (β −259 mm^3^, 95% CI −478 to −41 mm^3^, *P* = 0.02) and anterior femoroacetabular (β −277 mm^3^, 95% CI −491 to −63 mm^3^, *P* = 0.01) sites. The presence of cartilage defects at any posterior site tended towards being associated with reduced femoral head cartilage volume, but these relationships did not reach statistical significance (*P* = 0.08 to 0.11). In the central region, defects in the central superolateral, but not central inferomedial regions of the femoral head were associated with reduced femoral head cartilage volume (β −283 mm^3^, 95% CI −460 to −106 mm^3^, *P* = 0.002).

The associations between hip BMLs and femoral head cartilage volume in people with no diagnosed hip OA are shown in Table 3. After adjusting for age, gender, BMI and femoral head bone area, reduced femoral head cartilage volume was associated with the presence of BMLs at the anterior femoral head (β −684 mm^3^, 95% CI −1260 to −108 mm^3^, *P* = 0.02), and tended toward significance at the anterior acetabulum (*P* = 0.08) and femoroacetabulum (*P* = 0.05). The presence of BMLs in the posterior regions was not significantly associated with a reduction in femoral head cartilage volume. Central superolateral BMLs at any of the femoral head (β −610 mm^3^, 95% CI −990 to −229 mm^3^, *P* = 0.002), acetabulum (β −250 mm^3^, 95% CI −557 to −123 mm^3^, *P* = 0.003) or femoroacetabular sites (β −373 mm^3^, 95% CI −583 to −164 mm^3^, *P* = 0.001) were associated with reduced femoral head cartilage volume. No significant association was observed for central inferomedial BMLs.

## Discussion

Compared with community-based adults with no diagnosed hip OA, people with diagnosed hip OA have lower femoral head cartilage volume and a higher prevalence of cartilage defects and BMLs. For people with no diagnosed hip OA, both BMLs and cartilage defects in the anterior femoral head and central superolateral hip are associated with decrease hip joint cartilage volume, while BMLs and cartilage defects of the inferomedial femur are not. Cartilage lesions of the anterior acetabulum and anterior femoroacetabular region are also associated with decreased cartilage volume. These data demonstrate that cartilage defects and BMLs present in the anterior and central superolateral hip are associated with decreased cartilage volume, which is seen in hip OA, and may represent early structural changes of OA.

In this study we found that those with diagnosed hip OA had significantly less femoral head cartilage than those without. These findings are consistent with a previous study that showed that reduced femoral head cartilage volume was associated with more severe radiographic joint space narrowing [12]. It has been shown that there is a 13% mean reduction in femoral head cartilage volume, or a 9% mean reduction in cartilage thickness, with each increase in grade of radiographic joint space narrowing [12]. In our study, people with diagnosed hip OA had approximately half the volume of femoral head cartilage as people without, after adjusting for age, gender, BMI and femoral head bone area.

There are limited data on the prevalence of cartilage defects and BMLs in people with or without hip OA. Although previous MRI studies have attempted to characterise the prevalence of regional structural abnormalities [13,14], these have been limited by a small sample size and to our knowledge, have not provided regional prevalence data. The current study is the first to have documented prevalence data for regional structural abnormalities. Kumar *et al*. demonstrated that cartilage defects and BMLs were associated with greater self-reported pain and disability, and that acetabular cartilage defects were more common in people with radiographic hip OA than those without [13]. We have extended this work and demonstrated that compared with people with no diagnosed hip OA, cartilage defects and BMLs are generally more common in all regions of the OA hip joint, excluding the central inferomedial region.

We aimed to determine the relationships between hip structures in those without diagnosed hip OA to help better understand the pathogenesis of early hip OA. Thus, we excluded participants with diagnosed hip OA. Such analyses have not previously been performed. Using the analogy of the knee where it has been shown that the presence of cartilage defects predict cartilage volume loss and joint replacement surgery [18,19], we examined the association between regional cartilage defects and femoral head cartilage volume. Our data show that in people without diagnosed hip OA, cartilage defects in the anterior region of any one of the femoral head, acetabulum or combined femoroacetabular regions are associated with reduced femoral head cartilage volume. Similarly, cartilage defects in the central superolateral region of the femoral head are associated with reduced femoral head cartilage volume. Although cartilage defects were more prevalent in the posterior and central inferomedial regions than the anterior and central superolateral regions, it was only cartilage defects located in the anterior and central superolateral regions that were significantly associated with reduced femoral head cartilage volume. This provides the first evidence that cartilage defects in the anterior and central superolateral femoral head regions may be associated with early structural changes of hip OA.

At the knee, BMLs have been associated with pain, cartilage loss and joint replacement [2,6]. Little is known about BMLs at the hip. In people with no diagnosed hip OA, we have demonstrated that BMLs in the central superolateral region of the femoral head, acetabulum and femoroacetabular regions were all significantly associated with reduced femoral head cartilage volume. Likewise, a BML in the anterior region of the femoral head was associated with reduced femoral head cartilage volume. Nevertheless, it is important to acknowledge that there was a low prevalence of BMLs in the anterior femoral head in people without diagnosed hip OA (2.4%). However, the number of people with BMLs in the anterior femoroacetabular region was considerably higher (20.4%), and the association between anterior femoroacetabular BMLs and reduced femoral head cartilage volume still tended toward significance (*P* = 0.05).

This is the first study to examine the associations between the presence of cartilage defects and BMLs seen on MRI and femoral head cartilage volume in people with no diagnosed hip OA. A unifying theme to emerge from these data is the location-specific importance of BMLs and cartilage defects. Although a previous study attempted to distinguish differences in the locations of MRI lesions, a small sample size (n = 90) precluded analyses [13] and the authors noted that future studies are needed to evaluate the effect of the location of structural lesions. Another study of an even smaller sample (n = 50) examined a host of MRI determined structural abnormalities in a region-specific manner, but did not provide any regional prevalence data [14]. We have addressed this issue by providing prevalence data and in addition, demonstrated that for people without hip OA, cartilage defects and BMLs in the anterior and central superolateral regions were associated with reduced femoral head cartilage volume. In a previous study, cartilage defects in the anterior and superior regions of both femur and acetabulum were associated with worse self-reported disability [13]. Such lesions may therefore have the propensity to modify femoral head cartilage volume and symptoms, and determining risk factors for such lesions could provide an important therapeutic target to help reduce the onset or slow the progression of hip OA. Such claims will however need investigation in future studies. Nevertheless, the location specific importance of structural abnormalities at the hip joint may suggest that local biomechanical risk factors are important in their pathogenesis. Femoroacetabular impingement (FAI), a major cause of early hip OA, is a biomechanical impingement of the hip joint contributed to by either or both an aspherical femoral head (cam deformity) or focal or generalised acetabular over-coverage (pincer deformity), acting as a physical impediment to joint range of movement. Several smaller studies demonstrated that the cartilage damage observed among people with cam deformity was in a similar location to the damage we observed in the anterior and central superolateral regions of the hip [20–22].

This study had several limitations. The cross-sectional nature of this study precludes any causal effect between the role of variables such as cartilage defects and BMLs in the development of hip OA to be identified. Longitudinal studies will be required to determine these relationships. Although only a modest number of people with diagnosed hip OA were examined in this study, we noted a number of significant differences between people with and without diagnosed hip OA, suggesting that this study was not underpowered to detect these differences. Participants with diagnosed hip OA were not matched with participants without diagnosed hip OA. Thus participants with diagnosed hip OA were significantly younger than those without a diagnosis of hip OA. To best account for these differences, we have adjusted for age in all the analyses. However, if, as at the knee, cartilage volume tends to reduce with increasing age, the power of the study to detect a difference between those with and without hip OA would have been limited. Despite this potential limitation, we were able to demonstrate significant reduction in cartilage volume in those with hip OA. People with no diagnosed hip OA did not have radiographs performed in this study. Although some participants may have had early radiographic OA, they did not have sufficient symptoms to seek medical diagnosis or intervention. Moreover, the potential for some participants with early radiographic hip OA to have been included in the group with no diagnosed hip OA is likely to have underestimated any differences we observed secondary to non-differential misclassification. A further limitation of this study is that we did not have information on participants’ medications. However as no pharmacological agent has been shown to modify hip structure, failure to adjust for medication is unlikely to have been a significant confounder to the results of this study. Finally, it has been notoriously difficult to assess structural changes at the hip joint using MRI in epidemiological studies. Our division of the anterior, central and posterior regions was adapted from methods used in previously published works with smaller sample sizes [13,14]. We extended these works, providing the first regional prevalence data and evidence documenting the importance of the anatomical distribution of cartilage defects and BMLs at the hip. As it has been noted that the central inferomedial region of the acetabulum is not covered by cartilage, as in prior studies using MRI [13] and arthroscopy [17], it is not possible to score cartilage there.

## Conclusion

Reduced femoral head cartilage volume and increased prevalence of cartilage defects and BMLs are seen in people with diagnosed hip OA compared with those without a diagnosis of hip OA. Furthermore, for people without diagnosed hip OA, cartilage defects and BMLs in the anterior and central superolateral regions of the hip are associated with reduced femoral head cartilage volume. These data suggest that structural MRI abnormalities are present prior to clinical hip OA, and that cartilage defects and BMLs in the anterior and central superolateral regions identify early structural changes of hip OA and may be potential therapeutic targets for the prevention and early treatment of hip OA.

## Abbreviations

BMI: body mass index
BML: bone marrow lesion
CV: coefficient of variation
ICC: intra-class correlation coefficient
MCCS: Melbourne Collaborative Cohort Study
MRI: magnetic resonance imaging
OA: osteoarthritis
